# Relationship between Occupational Stress, 5-HT2A Receptor Polymorphisms and Mental Health in Petroleum Workers in the Xinjiang Arid Desert: A Cross-Sectional Study

**DOI:** 10.3390/ijerph14040402

**Published:** 2017-04-10

**Authors:** Ting Jiang, Hua Ge, Jian Sun, Rong Li, Rui Han, Jiwen Liu

**Affiliations:** 1 Department of Public Health, Xinjiang Medical University, Urumqi 830011, China; jt5583@126.com (T.J.); gehua2710@sina.com (H.G.); lurong_128@126.com (R.L.); 2 The Sixth Affiliated Hospital of Xinjiang Medical University, Urumqi 830011, China; sjt3997@163.com; 3 The First Affiliated Hospital, School of Medicine, Shihezi University, Shihezi 832008, China; shihaohua789@163.com

**Keywords:** 5-HT2RA, mental health, occupational stress, petroleum workers

## Abstract

At present, there is growing interest in research examining the relationship between occupational stress and mental health. Owing to the socioeconomic impact of occupational stress and the unique environment of petroleum workers in Xinjiang, a cross-sectional study was carried out between April and December 2015 to investigate the relationship between occupational stress, 5-hydroxytryptamine receptor (5-HTR2A) genotype, and mental health. A total of 1485 workers were selected. The Symptom Checklist 90 was used to assess nine classes of psychological symptoms. Work-related stressors were evaluated using the Occupational Stress Inventory-Revised Edition. Levels of 5-HTR2A (the Tl02C and A-1438G single nucleotide polymorphism in the 5-HTR2A gene) were measured by polymerase chain reaction and restriction fragment length polymorphism (PCR-RFLP). The findings of the present study revealed a high prevalence rate of mental health problems (40.29%) in petroleum workers stationed in the arid desert, and suggested a strong correlation between occupational stress and mental health. The TC and CC genotype of Tl02C were found to be protective factors against mental health problems (odds ratio (OR) = 0.455, 95% confidence interval (CI): = 0.269–0.771, odds ratio (OR) = 0.340, 95% confidence interval (CI): 0.162–0.716). AG and GG genotype of A-1438G [odds ratio (OR) 1 = 2.729, 95% confidence interval (CI): 1.433–5.195; odds ratio (OR) 2 = 2.480, 95% confidence interval (CI): 1.221–5.037] were revealed as risk factors. These data provide evidence that occupational stress and 5-HTR2A gene polymorphism contributes to the incidence of mental health problems.

## 1. Introduction

Due to the rapid development of society, science, technology, and the organization of the labour process as well as changes to ways of life, occupational stress has been shown to be an important social determinant of health that negatively impacts workers [1,2]. Psychological health is “a state of wellbeing in which the individual realizes his or her own abilities, can cope with the normal stresses of life, can work productively and fruitfully, and is able to make a contribution to his or her community” [3]. There is increased interest in research examining the relationship between occupational stress and mental health [4,5,6,7,8].

Some research has shown that stress significantly contributes to the development of mental illness [9,10], and that the higher the levels of stress, the higher the incidence of mental illness [11]. Furthermore, psychological distress is recognized as a strong predictor of the onset of a major depressive episode [12]. An et al. [13] have also highlighted that if there is no reduction in severe stressors, a further deterioration in mental health will ensue.

Individuals exhibit a broad range of nervous system responses which may be partly attributed to genetic factors. Adrenal responses to stress—reflecting deviations from normal physiological or psychological set points—demonstrate area major function of the hypothalamic-pituitary-adrenal (HPA) axis in general adaptation syndrome [14]. This axis is one of the body’s primary hormonal systems for responding to stress and the first to respond to social stressors [15]. Moreover, the basal activity and reactivity of the hypothalamic-pituitary-adrenal (HPA) axis is modulated by and in turn modulates neurotransmitters such as serotonin (5-hydroxytryptamine, 5-HT) [16,17,18]. The 5HTR2A gene is located onchromosome 13 (13q14-q21); it spans 20 kb and contains 3 exons; more than 200 SNPs have been identified along the gene [19,20]. Genetics can predict 5-HT system activation, given its relationship to strain [21]. 5-HT is an important monoamine neurotransmitter in the body that may mediate anxiety, depression, and other related mood disorders. Previous studies have suggested an association between 5-HT receptor gene polymorphism and depression [22,23,24]. Mainly, the polymorphisms A-1438G (rs6311), and T102C (rs6313) have been studied in relation to mental health. Mental health may also be correlated with T102C and A-1438G gene polymorphism in the 5-HTR2A [25,26].

Petroleum workers employed by the Xinjiang Field Petroleum Administration Bureau are a unique category of professionals. These employees have long working hours, engage in shift work every six months, and their tasks are very arduous. Workplaces are situated in arid deserts where employees are subjected to isolation, extreme weather, and heavy sand storms. Several studies have reported that mental and physical health are affected by natural conditions including the monotonous lifestyle of arid deserts and other harsh environments [27,28]. Due to the socioeconomic impact of occupational stress [29], the majority of research studies have focused on white-collar workers [30,31] although few studies have examined the relationship between occupational stress, 5-HTR2A polymorphism, and mental health in petroleum workers stationed in arid deserts [32,33]. This research conducted a cross-sectional study of petroleum workers in Xinjiang to investigate: (1) the relationship between occupational stress and mental health; and (2) the relationship between 5-HTR2A polymorphism and mental health.

## 2. Methods

### 2.1. Participants

This current research was a cross sectional study which performed between April to December 2015. In 2015, Petroleum workers were selected from the Xinjiang Petroleum Administration Bureau of China National Petroleum Corporation in Karamay City, Xinjiang Province, China. Using a three-stage stratified sampling method, five operating areas, five production factories, and five exploration and development companies were selected based on the Chinese Standard Industrial Classification of the nature of the companies. Then, based on the size of the companies, we randomly selected two large company (>1000 workers) and one small company (<1000 workers) from selected operating areas; one large company (>600 workers) and one small company (<600 workers) from selected production factories; and one large (>200 workers) and two small (<200 workers) exploration and development companies. Finally, for operating areas, we selected 600 workers from large companies, 345 from small companies; for production factories, we selected 390 and 160 workers from large and small companies, respectively; for exploration and development companies, we selected 170 and 90 workers from large and small companies, respectively. Workers were selected by applying computer-generated random numbers to the managers’ lists of employees. In the course of their work, the participants engaged in geophysical prospecting, drilling, testing, downhole operation, transferring oil, etc. All participants have a period of employment longer than one year. Those who had a prior history of mental illness such as depression, anxiety disorder, bipolar disorder, schizophrenia etc., were excluded from the study. All participants provided written informed consent. Ultimately, 1485 workers were selected to participate in this current research, and their demographic and occupational data we obtained from worker information system, which was approved by Xinjiang Petroleum Administration Bureau of China National Petroleum Corporation. This study was approved by the Ethics Committee of the First Teaching Hospital at Xinjiang Medical University, Urumqi, China. (Identification Code: 2015008).

### 2.2. The Self-Report Symptom Inventory: Symptom Checklist 90 Revised (SCL-90)

Mental health was measured using the Chinese SCL-90-R [34]. The SCL-90-R is a 90-item self-report symptom inventory, multidimensional in nature, and oriented toward the measurement of psychopathology. Each of the 90 items is rated on a 5-point scale of distress, ranging from 1 to 5 was used: 1 = no, 2 = mild, 3 = moderate, 4 = quite serious, 5 = serious. The SCL-90-R is scored on the following nine primary symptom dimensions: somatisation; obsessive-compulsive; interpersonal sensitivity; anger-hostility; depression; anxiety; paranoid ideation; phobic anxiety; and psychoticism [35]. Higher scores of SCL-90 indicate more severe psychological symptoms which may result in higher prevalence of workers with mental health problems depending on the cut-off value, A total score exceeding 160 points on the SCL-90 was used to measure the presence of mental health problems [36].

### 2.3. Occupational Stress Inventory Revised Edition (OSI-R)

Occupational Stress were measured using the Occupational Role Questionnaire subscales from the Chinese version of the OSI-R [37]. The OSI-R is a reliable and valid method for measuring stressors in different occupational populations throughout China [38,39]. The OSI-R consists of three subscales: Occupational Roles Questionnaire (ORQ), Personal Strain Questionnaire (PSQ), and Personal Resources Questionnaire (PRQ). The ORQ included six subscales (i.e., Role Overload, Role Insufficiency, Role Ambiguity, Role Boundary, Responsibility, and Physical Environment) and was used to assess work-related stressors. The PSQ included four subscales (i.e., Vocational Strain, Psychological Strain, Interpersonal Strain, and Physical Strain) and was used to assess levels of personal strain. The PRQ included four subscales (i.e., Recreation, Self-Care, Social Support, and Rational Coping) and was used to assess coping resources. A scale ranging from 1 to 5 was used to assess occupational stress. While higher scores for the ORQ and PSQ indicated higher stress levels, higher scores for the PRQ corresponded with lower stress levels and a greater ability to cope with stress.

### 2.4. Quality Control of Questionnaires

All questionnaires were provided to participants and immediately collected in the activity centre. The questionnaires were completed anonymously within 15 min. As the initialization of the survey, participants are instructed to be aware of how they should complete questionnaires, realize the significance of the study, and ensure that the participants cooperated well so that they could truthfully and accurately complete each questionnaire item. Throughout this time, two experienced psychologists and two officers engaged with participants to ensure cooperation with the research team as well as to safeguard the authenticity and efficacy of the questionnaire responses. During the survey, and after having recorded the results, the completed questionnaires were comprehensively reviewed by two researchers in order to examine any uncertainty concerning the responses, to correct errors, and to address any omissions in questionnaire responses.

### 2.5. Genetic Biomarkers

Thirty percent of the petroleum workers who had correctly completed their questionnaires were selected from the mental health problems group through the use of random sampling to determine the polymorphisms of 5-HTR2A, and normal group which is determined as normal condition depending on SCL-90 matched with mental health problems group 1:1 based on the gender, ethnicity, age and type of work to determine the polymorphisms of 5-HTR2A. Fasting blood samples (5 mL) were obtained as part of the health examination, and were anonymised. Prior to the health examination, participants were requested to abstain from a high-fat diet and alcohol for 3 days. Blood samples were placed in heparin-anticoagulant tubes and centrifuged at 3000 rpm for 10 min at 4 °C. Plasma was collected and stored at –20 °C. Levels of (5-HTR2A) were measured by polymerase chain reaction and restriction fragment length polymorphism (PCR-RFLP).

### 2.6. Statistical Analysis

Two researchers independently recorded the results in the database and a consistency test was conducted. Data were analysed using SPSS for Windows v.17.0 software (SPSS Inc., Chicago, IL, USA). Continuous data were presented as mean ± standard deviation and compared using the Student’s *t*-test. Categorical data were presented as frequencies, and analysed using the chi-squared (χ^2^) test. The χ^2^ test was used evaluate Hardy-Weinberg equilibrium (HWE). Hardy-Weinberg equilibrium is assumed when observed genotype and allele frequencies between mental health problems and normal group are in equilibrium in a population. To examine the risk associated with mental health problems, odds ratios (OR) and 95% confidence intervals (CIs) were examined using multivariate logistic regression. *p*-values < 0.05 were considered statistically significant.

## 3. Results

### 3.1. Characteristics of the Participants

One thousand four hundred and eighty-five questionnaires were distributed, and 1380 were collected and verified to be satisfactory, giving a total retrieval rate of 92.9%. At the end of survey, 556 participants were deemed to exhibit a mental health problems. Thus, the prevalence rate was 40.29%. While a statistically significant difference was found between the mental health problems group and normal group with regard to alcohol consumption (*p* = 0.009), there were no statistically significant differences for all other variables (gender, age group, year, ethnicity, working years, type of work, shift, professional title, educational level, marital status, monthly family income and smoking) (*p* > 0.05) (Table 1).

The participants’ total score for mental health was 161.79 ± 55.27. The total score as well as the individual scores associated with each of the nine variables were found to be higher than the national norm (Table 2).

### 3.2. Occupational Stress Variables between the Two Groups

The degree of Role Overload ((*t* = 5.571, *p* < 0.001), Role Insufficiency (*t* = 4.299, *p* < 0.001), Role Ambiguity (*t* = 2.569, *p* < 0.001), Role Boundary (*t* = 4.640, *p* < 0.001), Responsibility (*t* = 5.097, *p* < 0.001), Physical Environment (*t* = 2.663, *p* < 0.001), Vocational Strain Reaction (*t* = 8.765, *p* < 0.001), Psychological Strain Reaction (*t* = 11.220, *p* < 0.001), Interpersonal Strain Reaction (*t* = 7.759, *p* < 0.001), and Physical Strain Reaction (*t* = 13.549, *p* < 0.001) were significantly higher in the mental health problems group than in the normal group which are determined as normal condition depending on SCL-90 . 

The degree of Recreation (*t* = −2.128, *p* < 0.034), Social Support (*t* = −3.368, *p* = 0.001), and Rational Coping (*p* = 0.001) were significantly lower in the mental health problems group than in the health control group (Table 3).

### 3.3. Genetic Balance Check

The χ^2^ test was used to evaluate Hardy–Weinberg equilibrium, and suggested a better match between actual values and expected values with regard to 5-HTR2A T102C and A-1438G polymorphism in the sample population (*p* > 0.05). This finding suggests that the research sample population is within the genetic equilibrium state, and is representative of the general population (Table 4).

### 3.4. Comparison of T102C, the A-1438G Polymorphism between the Two Groups and the Risk of Mental Health Problems Associated with This Genotype

The results of this study shows a statistically difference between the mental health problems group and the normal group in terms of the T102C, A-1438G polymorphism (*p* < 0.05). There was no statistically difference between the two groups in the allelic gene of T102C and A-1438G (*p* > 0.05) (Table 5).

The association between Tl02C, A-1438G polymorphism and mental health problems was examined using multivariate logistic regression analysis. With reference to the TT genotype of T102C gene polymorphism, the results revealed that carrying the TC and CC genotypes acts as a protective factor against mental health problems (OR = 0.455, 95% CI = 0.269–0.771; OR = 0.340, 95% CI = 0.162–0.716). With regard to the AA genotype of the A-1438G gene polymorphism, carrying the AG genotype increases the risk of mental health problems in AA genotype by 2.749 times (95% CI = 1.488–5.080). Carrying the GG genotype increases the risk of mental health problems in AA genotype by 2.654 times (95% CI = 1.350–5.218). In adjusting Vocational Strain, Psychological Strain, Physical Strain, Social Support, and Rational Coping, T102C gene polymorphism of the TC and CC genotypes reduces the risk of mental health problems (OR = 0.443, 95% CI = 0.255–0.770, OR = 0.360, 95% CI = 0.164–0.790). With reference to A-1438G gene polymorphism, carrying AG and GG genotypes increases the risk of health problems (OR1 = 2.729, 95% CI = 1.433–5.195, OR2 = 2.480, 95% CI = 1.221–5.037) (Table 6).

## 4. Discussion

This study investigated the relationship between occupational stress, 5-HT2A receptor polymorphisms and mental health in petroleum workers in the Xinjiang arid desert. As a complex and chronic disease, mental disorder is affected by many factors. Some studies [40,41,42] have found that higher stress levels were more prevalent among females than males. However, this current research found that incidence of mental health problems is no statistically significant differences with regard to gender differences. This finding may be attributed to the unique work environment, the various tasks carried out by employees as well as the diverse methods that may be used for mental health screening.

The participants’ alcohol consumption proved to be a statistically significant variable in mental health problems. Abstinence from alcohol consumption was found to be a protective factor against mental health problems. This finding is consistent with the results of Lacey et al. [43]. Petroleum workers spend prolonged periods of time in a unique environment, and are often subjected to noise and heavy workloads. With few recreational activities available, these employees may be attracted to consuming alcohol in an effort to relieve stress, which only increases alcohol dependency, inevitably leading to the onset of depression, anxiety and other psychological disorders.

To determine the incidence of mental health problems, this current study used a total score of >160 points, as measured using the SCL-90-R. The survey featured 556 participants. The incidence rate was found to be 40.29%. The total score as measured using the SCL-90-R was 161.79 + 55.27 points. While this result is higher than the total score found in the Zhang et al. study [44] which examined the mental health of petroleum workers, this score is also higher than the national norm. Thus, petroleum workers exhibit a higher incidence of mental health problems. This finding may be related to long-term contact with occupationally harmful factors encountered in the course of the work such as bad weather, dust, noise, and crude oil. In addition, emergencies and unforeseen factors may occur without warning. These factors may contribute to the onset and development of mental health problems in petroleum workers, such as anxiety, loneliness, agitation, and may increase the risk of workplace accidents.

Significant differences were found between the mental health problems group and normal group with regard to work-related stressors and personal stress. This results suggests that these variables may have positive correlation with the incidence of mental health problems. Relevant epidemiological studies have highlighted stress as a risk factor for mental health problems [45], showing a strong correlation between stress and mental health problems. Progressively high levels of stress correlate with an increased risk of developing a mental health problems [46]. Statistically significant differences were found between the two groups with regard to Recreation, Social Support and Rational Coping, suggesting that this factor plays a significant role in an individual’s ability to cope with mental health problem. This results offer an insight into the factors associated with mental health, and underscore the need to establish policies to minimise occupational stress in order to protect the health of petroleum workers in arid deserts.

The results of this current study revealed a statistically significant difference between the mental health problems group and normal groups with regard to the T102C and A-1438G genotypes, suggesting that T102C and A-1438G genotypes of the 5-HTR2A genewere associated with the incidenceof mental health problems. Although this finding was consistent with the results of some previous research [47,48], other studies [49,50] obtained the opposite result, which may be attributed to the use of different sample populations or the influence of regional differences. Logistic regression analysis was used to examine the relationship between T102C and A-1438G genotypes and the risk of mental health problems. With regard to the T102C gene, and after having adjusted for some occupational stress factors, the results suggest that carrying the TC and CC genotype as opposed to the TT genotype may reduce the risk of mental health problems. Furthermore, the C allele may be a protective factor against mental health problems. The Zhang et al. study [51] examined the relationship between 5-HT2A receptor gene T102C polymorphism and grey matter density in patients with depression. The results revealed that the C allele may be a mediating factor as alleles within the human brain structure may increase susceptibility to regional depression. However, in normal brain structure, C alleles exert no obvious effect. Similarly, the Chen et al. study [52] concluded that the C allele is a protective factor against depression following a stroke. With regard to the A-1438G gene, carrying AG and GG genotypes as opposed to the AA genotype may increase the risk of mental health problems. Zhao et al. [24] concluded that the risk of carrying GG genotype is as 1.20 times high as that of AA + AG genotype (CI = 1.02–1.43). Additionally, a study conducted by Kim et al. [53] obtained a similar conclusion. These findings suggest that the T102C C genotype and A-1438G G genotype may, to a certain extent, affect mental health problems, and that factors inherent to occupational stress may furthermore influence the onset and development of mental illness.

There were some limitations to the present study. Due to the descriptive study design, causal relationship cannot be established in general. Second, the sample size was small. Participants were recruited from a single administration bureau in Xinjiang, which might not be representative of the entire population of petroleum workers in China. Third, we used a self-report questionnaire to measure the prevalence of mental health problems, which may have yielded recall/report bias. Finally, this study investigated a minimal number of polymorphisms that are correlated with stress. However, it is possible that other polymorphisms of the 5-HTR2A gene influence susceptibility to mental health problems. In the future, the researchers aim to undertake a cohort study to investigate occupational stress-induced mental problems in individuals exposed to the Xinjiang arid desert environment. A study of this nature could provide additional evidence that may highlight the importance of understanding occupational stress and mental health problems in petroleum workers.

## 5. Conclusions

In conclusion, the results of this study revealed a high incidence rate of mental health problems in petroleum workers stationed in the arid desert, and furthermore suggested a strong correlation between occupational stress and mental health problems. TC and CC genotypes of 5-HTR2A were found to be protective factors against the onset and development of mental health problems, whereas AG and GG genotypes were identified as risk factors.

## Figures and Tables

**Table 1:** Characteristics of the sample.

Variables		All Participants (1380)		Mental Disorder		χ^2^	*p*
		*n*	%	*n*	%		
Gender	Male	745	53.99	304	40.81	0.179	0.672
	Female	635	46.01	252	39.69		
Age group, year	≤30	199	14.42	80	40.20	0.978	0.522
	>30	1181	85.58	476	40.30		
Ethnicity	Han	1107	80.22	446	40.29	0.000	0.999
	Minority	273	19.78	110	40.29		
Working years	≤15	412	29.86	171	41.50	0.291	0.589
	>15	968	70.14	385	39.77		
Type of work	Oil transportation	548	39.71	228	41.60	0.53	0.467
	Extract oil	405	29.35	144	35.56		
	Stoker hot note work	427	30.94	184	43.09		
Shift	No	529	38.33	249	41.57	2.706	0.100
	Yes	851	61.67	307	39.30		
Professional title	Primary and secondary	776	56.23	311	40.08	0.36	0.548
	Vice-senior and Senior	604	43.77	245	40.56		
Educational Level	Associate’s degree or below	563	40.8	222	39.43	5.561	0.062
	Bachelor’s degree or higher	817	59.2	334	40.88		
Marital status	Single	121	8.77	45	37.19	0.033	0.855
	Married	1259	91.23	511	40.58		
Monthly family income	≤3000	309	22.39	112	36.25	0.72	0.396
	>3000	1071	77.61	444	41.56		
Smoking	Yes	445	32.25	175	39.32	0.254	0.614
	No	935	67.75	381	40.75		
Drinking	Yes	624	45.22	275	44.07	6.678	0.009
	No	756	54.78	281	37.17		

**Table 2:** Assessment results of each component and the total score of Symptom Checklist 90 (SCL-90) $\left(\bar{\chi }\pm s\right)$.

Variables	SCL-90 Score	Reference Range of China
Total score	161.79 ± 55.27	129.96 ± 38.76
Somatization	1.92 ± 0.68	1.37 ± 0.48
Obsessive–compulsive	1.97 ± 0.70	1.62 ± 0.58
Interpersonal sensitivity	1.79 ± 0.65	1.65 ± 0.61
Depression	1.80 ± 0.67	1.50 ± 0.59
Anxiety	1.77 ± 0.65	1.39 ± 0.43
Hostility	1.76 ± 0.66	1.48 ± 0.56
Phobia	1.65 ± 0.66	1.23 ± 0.41
Paranoid	1.72 ± 0.67	1.43 ± 0.57
Psychotic symptoms	1.65 ± 0.64	1.29 ± 0.42

**Table 3:** Comparison of occupational stress variables between the two groups ($\bar{\chi }\pm s$).

Variables	Mental Health Problems	Normal	*t*	*p*
Role overload	28.38 ± 6.87	26.26 ± 6.92	5.571	<0.001
Role insufficiency	31.03 ± 7.77	29.24 ± 7.33	4.299	<0.001
Role ambiguity	32.07 ± 7.05	31.09 ± 6.87	2.569	<0.001
Role boundary	27.80 ± 6.27	26.17 ± 6.50	4.640	<0.001
Responsibility	25.96 ± 7.13	23.95 ± 7.18	5.097	<0.001
Physical environment	32.17 ± 7.85	31.02 ± 7.90	2.663	<0.001
Vocational strain	26.85 ± 6.27	23.92 ± 5.96	8.765	<0.001
Psychological strain	28.25 ± 6.23	24.34 ± 6.42	11.220	<0.001
Interpersonal strain	28.22 ± 5.47	25.82 ± 5.78	7.759	<0.001
Physical strain	27.80 ± 5.92	23.38 ± 5.97	13.549	<0.001
Recreation	27.85 ± 6.50	28.65 ± 7.10	−2.128	0.034
Self-care	28.73 ± 6.55	29.18 ± 8.87	−1.214	0.225
Social support (SS)	31.24 ± 7.29	32.74 ± 8.62	−3.368	0.001
Rational coping	28.94 ± 6.45	30.23 ± 7.50	−3.304	0.001

**Table 4:** Hardy-Weinberg equilibrium test.

Genotype	Actual Values	Expected Value	χ^2^	*p*
T102C				
TT	108	120.95	3.589	0.166
TC	194	169.33		
CC	48	58.84		
A-1438G				
AA	73	74.61	0.205	0.902
AG	181	174.21		
GG	96	99.36		

**Table 5:** The distribution of T102C, A-1438G genotypes and alleles *n* (%).

Genotype	Mental Health Problems	Normal	χ^2^	*p*
T102C				
TT	63 (36.00%)	45 (25.71%)	6.186	0.045
TC	94 (53.71%)	100 (57.14%)		
CC	18 (10.29%)	30 (17.15%)		
T	220 (62.86%)	190 (54.29%)	1.726	0.189
C	140 (37.14%)	148 (45.71%)		
A-1438G				
AA	27 (15.43%)	46 (26.28%)	6.584	0.037
AG	99 (56.57%)	82 (46.86%)		
GG	49 (28.00%)	47 (26.86%)		
A	153 (43.71%)	174 (49.14%)	1.991	0.158
G	197 (56.29%)	181 (50.86%)		

**Table 6:** The risk of mental health problems.

Genotype		OR (95% CI)	*p*	OR * (95% CI)	*p*
T102C	TT	1	-	1	-
	TC	0.455 (0.269–0.771) *	0.027	0.443 (0.255–0.770) *	0.027
	CC	0.340 (0.162–0.716) *	0.044	0.360 (0.164–0.790) *	0.043
A-1438G	AA	1	-	1	-
	AG	2.749 (1.488–5.080) *	0.032	2.729 (1.433–5.195) *	0.031
	GG	2.654 (1.350–5.218) *	0.031	2.480 (1.221–5.037) *	0.029

OR adjusted for the Vocational Strain, Psychological Strain, Physical Strain, Social Support and Rational Coping; * *p* < 0.05.
